# Infant Can Visually Differentiate the Fresh and Degraded Foods: Evidence From Fresh Cabbage Preference

**DOI:** 10.3389/fpsyg.2019.01553

**Published:** 2019-07-30

**Authors:** Jiale Yang, Katsunori Okajima, So Kanazawa, Masami K. Yamaguchi

**Affiliations:** ^1^Department of Life Sciences, The University of Tokyo, Tokyo, Japan; ^2^Japan Society for the Promotion of Science, Tokyo, Japan; ^3^Faculty of Environment and Information Sciences, Yokohama National University, Yokohama, Japan; ^4^Department of Psychology, Japan Women’s University, Kawasaki, Japan; ^5^Department of Psychology, Chuo University, Tokyo, Japan

**Keywords:** infant, freshness perception, visual preference, preferential looking, developmental perception

## Abstract

Perceiving and judging food quality is indispensable in daily life. The present study examined this ability’s development in infants during the early postnatal months. We tested if infants aged 5–8 months can discriminate different degree of freshness in cabbage, strawberry, carrot, and spinach. In Experiment 1, images of fresh and degraded vegetables were presented side by side; infants aged 7–8 months significantly preferred fresh over degraded cabbage images. In Experiments 2 and 3, infants aged 7–8 months maintained their preference when the images were achromatic, but no longer preferred the fresh cabbage images when pixels in those images were randomized. Given these results, we suggest that the ability to discriminate different degrees of freshness, at least for cabbage, develops at approximately 7–8 months of age, which is the time probably prior to taste learning.

## Introduction

Human visual perception develops rapidly during the first month of life. Infants aged less than 8 months can perceive visual attributes of objects, such as contrast, orientation, lightness, color, and 3D shape (for a review, see Braddick and Atkinson, 2011). Objects have numerous non-visual properties. Judging material and condition is equally important to judging color and shape. For instance, in a supermarket, most adults can quickly identify ripe or fresh fruit and vegetables without touching or tasting them. Visual cues allow us to evaluate foods’ freshness. Increasing research has examined visual freshness perception in humans and chimpanzees (Wada et al., 2010; Arce-Lopera et al., 2012, 2013; Murakoshi et al., 2013; Imura et al., 2016); however, freshness perception’s development after birth remains unclear.

To discriminate different degrees of food freshness, infants need to perceive the surface features (e.g., smooth, wet, soft) and utilize this information to infer its physical properties. However, inferring physical properties from optical features is not a simple task. The appearance of a surface is determined by the surface reflectance, the surface shape, and the lights in the environment. These reflectance properties can be defined by the bi-directional reflectance distribution function (BRDF) in optics. BRDF is a complex function, and it is impossible for vision systems to solve this function. Instead, recent studies in the domain of vision science demonstrated that similar reflectance properties have consistent structures, and a visual system appears to use image statistics (e.g., pixel intensity distributions, wavelet coefficient distributions, luminance distribution, and so on) to infer the surface properties (Adelson, 2001; Motoyoshi et al., 2007). Research examining visual freshness perception has found that degradation is correlated with changes in a stimulus images’ luminance distribution and that humans and chimpanzees can use these cues to evaluate the freshness of fruit, vegetables, and fish (Wada et al., 2010; Arce-Lopera et al., 2012, 2013, 2015; Murakoshi et al., 2013; Imura et al., 2016). A recent study also showed that chimpanzees are able to discriminate between various degrees of freshness based on the luminance distribution (Imura et al., 2016). On the other hand, a recent infant study revealed that 9-month olds showed sensitivities to natural image statistics (Balas and Woods, 2014). Moreover, infants aged 7–8 months appear to be able to perceive surface glossiness (Yang et al., 2011, 2015). These results indicate that infants at this age have the ability to detect image statistics and are able to use these visual cues to infer the properties of a surface. Therefore, it is possible that infants have a rudimentary ability to discriminate between different degrees of freshness using similar visual cues. It must be noted that the fact that infants can discriminate different degrees of freshness does not mean that infants understand the concept of “freshness,” because acquisition of concepts relies on the development of high-level cognitive functions. However, the grasping of the concept of “freshness” requires the fundamental visual ability to discriminate between different degrees of freshness. Although several studies have investigated how our food cognition develops and how early experience affects the development of food preferences, there is a lack of studies investigating how the ability to appropriately identify the quality of foods initially develops. The present study aimed at understanding how infants develop this rudimentary visual ability.

This study used the preferential looking paradigm to test if infants can discriminate between stimuli showing different degrees of freshness. Cabbage, strawberries, carrots, and spinach were used as the visual stimuli because they are common agricultural products in Japan. In Experiment 1, we tested if infants could discriminate between stimuli depicting fresh and degraded food items. In Experiments 2 and 3, we tested if infants’ preferences would persist the following randomization of the images’ pixels (to remove the surface texture of the food but preserve its color composition) or conversion of the image into grayscale (to remove the image’s color but preserve its depiction of the object).

## Experiment 1

**Figure 1:**
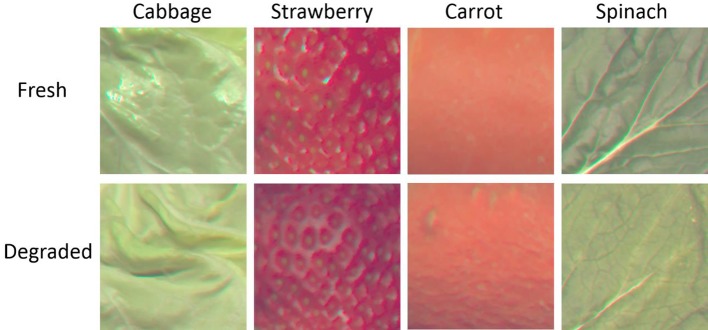
Stimuli used in Experiment 1.

In Experiment 1, we examined preferential looking at fresh food to test if infants can discriminate between images of fresh and degraded foods. We presented stimuli depicting fresh or degraded foods (Figure 1) side by side on a CRT monitor and observed which stimulus infants looked at longer during a fixed period.

### Method

#### Participants

Participants were 19 infants aged 5–6 months (nine males, 10 females; mean age = 168 days, range = 144–193 days), and 19 infants aged 7–8 months (10 males, nine females; mean age = 230 days, range = 208–252 days). Different participants were tested across the two age groups. An additional eight infants participated but were excluded from the analysis due to failure to complete the test (n = 4), side bias of >90% (n = 2), or low birth weight (n = 2). All participants were recruited through newspaper advertisements and were full term at birth and healthy during the experiment. None of the participants’ parents reported any family history of color deficiency. All experiments in present study were approved by the Ethics Committee of Chuo University and were conducted in accordance with the Declaration of Helsinki. Participants’ parents provided a written indication of informed consent.

#### Apparatus

The infant sat on his or her parent’s lap in the experimental booth during the experiment. A 21-inch color CRT monitor was located approximately 40 cm in front of the infant and displayed all stimuli (Sony GDM-F520). Participants’ looking behavior was recorded using a video camera located under the monitor. Behind the experimental booth, the experimenter observed participants’ behavior using a TV monitor and controlled the presented stimuli using a computer.

#### Stimuli

Samples of cabbage, carrot, spinach, and strawberry were randomly selected from a local market. To cause the food samples’ rapid deterioration, we put them in a controlled environment of 30°C temperature and 6% humidity. A 2D luminance and chromaticity analyzer (TOPCON UA1000) were used to measure the samples’ luminance and chromaticity every 30 min for several days. Subsequently, a fresh and a degraded food sample were selected. The fresh sample was selected at the first measurement (i.e., 0 h). The degraded sample was selected according to the author’s judgment that the sample was no longer appetizing. Degraded cabbage samples were selected at 8 h after the samples’ entry into the controlled environment. The strawberry, carrot, and spinach samples were selected at 72, 18, and 66 h, respectively. Color stimuli were created using a color management system to ensure exact reproduction of the sample’s luminance and chromatic data and cropped to square patches to eliminate global cues (e.g., the object’s size and shape; Figure 1). Table 1 shows the color difference and luminance difference between fresh and degraded images. No consistent change across foods was observed along degradation time. The stimuli’s viewing angle was 14.2° × 14.2°. Using the same stimuli in an adult experiment, Arce-Lopera et al. (2015) found that freshness rating decreased significantly depending on degradation time. Furthermore, chimpanzees were able to choose fresher texture stimuli of cabbage, which were similar to our stimuli (Imura et al., 2016). These results indicated that both adults and chimpanzees can distinguish between the fresh and degraded texture images by the perceived freshness.

**Table 1:** Color difference and luminance difference between fresh and degraded images.

	Color difference in CIE L*a*b* color space	Luminance difference (cd/m^2^)
Spinach	−3.53	−5.26
Cabbage	1.15	−5.04
Carrot	1.62	2.58
Strawberry	6.21	2.45

#### Procedure

Before the experiment, each infant’s parent was instructed not to look at the stimuli. To attract the participants’ attention, before each trial, a fixation figure and a short beep were presented to the participant. The experimenter started the trial after confirming that the infant was looking at the fixation figure and the parent complied the instruction. Each infant viewed four types of food, and each food was presented for two trials that had a fixed duration of 15 s. The position of the paired images for each food was reversed across two trials. The total eight trials were assigned to two blocks: each block was composed of four trials with four different types of food. The order of the presentation and the position of the paired images were randomized in each block for each infant. To confirm that the visual preference was not simply based on the glossiness difference of fresh and degraded images, eight adults (mean age = 27.6 years; SD = 1.8) rated the glossiness of fresh and degraded images using a 5-point scale over 32 trails (8 images × 4 ratings).

#### Data Coding and Analysis

For each trial, an observer who was blinded to the stimulus’ identity measured participants’ looking time offline. The observer selected one of three behavioral categories (i.e., left, right, or not looking) based on the video presentation of the trial, in real time. Data coding was continually performed during each trial. Twenty-nine percent of the looking time in each trial was recorded by a second trained observer. These reliability coding and the interrater reliability of two observers were calculated by intraclass correlation coefficient (ICC) using SPSS statistical package version 23 (ICC = 0.89 with 95% confident interval = 0.85–0.92).

### Results and Discussion

**Figure 2:**
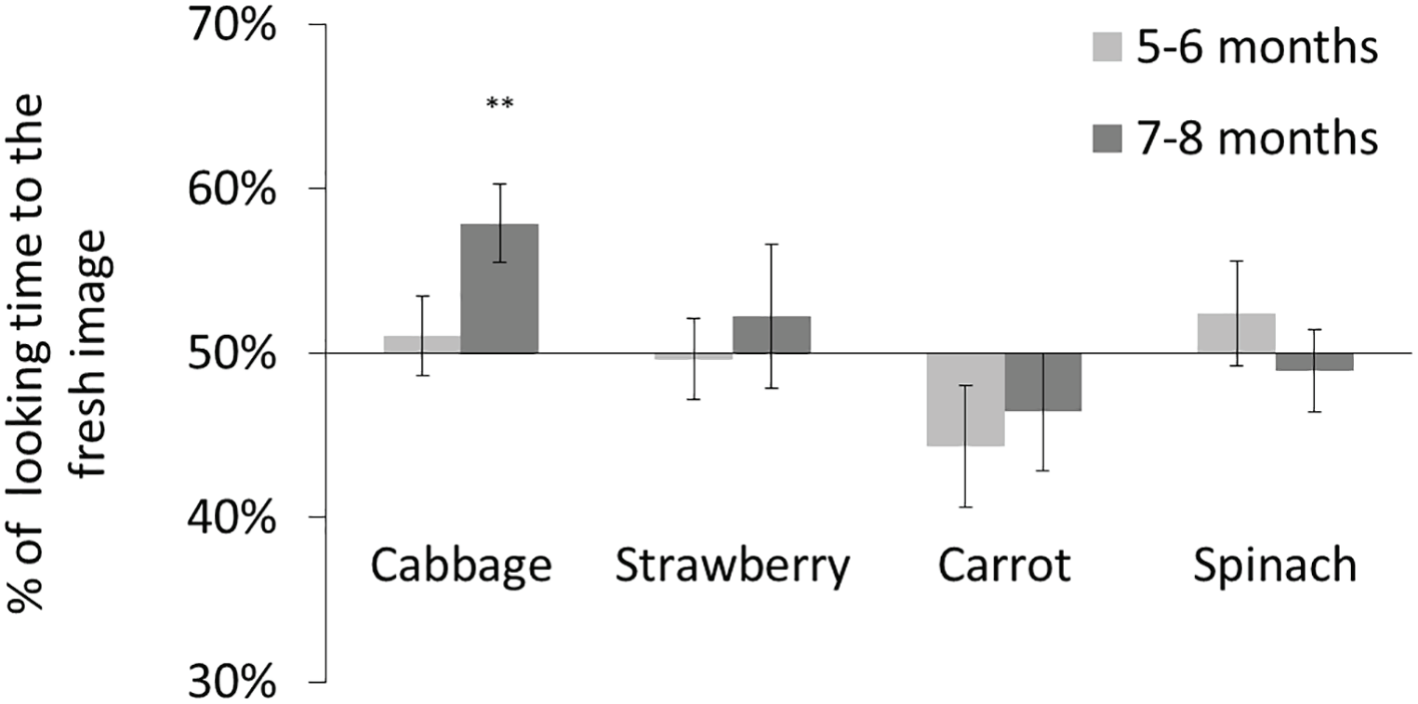
Result of Experiment 1. Mean percentage of looking time for fresh images. The error bars represent SE. Asterisks indicate the significance level of statistical differences: ^**^p < 0.01.

The mean total looking time over eight trials was 68.4 s (57.0% of the total trial duration) for participants aged 5–6 months and 68.0 s (56.7% of the total trial duration) for participants aged 6–8 months. We calculated a preference score for each participant (i.e., the ratio of time looking at the fresh image versus total looking time; Figure 2). We conducted a two-tailed *t* test comparing with chance (i.e., equal time spent looking at either stimulus) to test for significant preferences for fresh images in each age group. Participants aged 7–8 months preferred fresh cabbage stimuli [*t*(18) = 3.28, *p* < 0.01, Bonferroni-corrected, *d* = 0.75] but showed no other significant preferences [strawberry: *t*(18) = 0.65, ns; carrot: *t*(18) = 1.01, ns; spinach: *t*(18) = 0.41, ns]. Participants aged 5–6 months showed no significant preferences [cabbage: *t*(18) = 0.43, ns; strawberry: *t*(18) = 0.15, ns; carrot: *t*(18) = 1.53, ns; spinach: *t*(18) = 0.75, ns]. These results suggest a possibility that infants aged 7–8 months may develop an ability to discriminate fresh cabbage and degraded cabbage, whereas infants aged <6 months cannot.

**Figure 3:**
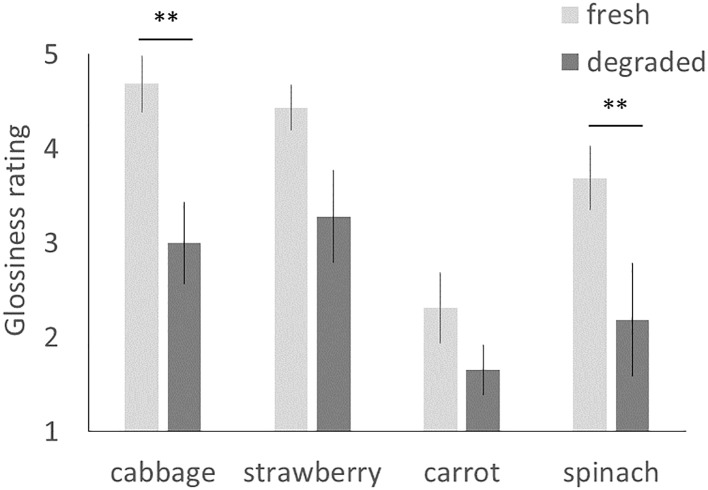
The average ratings for glossiness. The error bars represent SE. Asterisks indicate the significance level of statistical differences: ^**^p < 0.01.

One may argue that the preference for fresh cabbage image might stem from the higher glossiness perceived in the fresh cabbage image, because infants tend to look longer at the glossy object than at the matte object (Yang et al., 2011). We tested this possibility by calculating the difference of the glossiness ratings for each food pair. If the visual preference was based on the perceived glossiness difference, we would observe a correlation between the infants’ visual preference and the difference of the glossiness ratings. Figure 3 illustrates the difference of the glossiness ratings in each food pair. Bonferroni-adjusted *post hoc* comparisons indicated that glossiness ratings differed significantly between fresh and degraded cabbage and spinach [cabbage: *t*(7) = 6.30, *p* < 0.01, *d* = 2.27; spinach *t*(7) = 3.10, *p* < 0.01, *d* = 1.54] but not between fresh and degraded strawberries and carrots [strawberry: *t*(7) = 2.71, ns; carrot: *t*(7) = 2.54, ns]. Regarding the visual preference for the image pair, the infants only discriminated between fresh and degraded cabbage images. If the preference for fresh cabbage image was caused by the glossiness perception, we should observed preference for the fresh spinach image too. However, infants aged 7–8 months showed no preference for the fresh spinach image. These results indicated that discrimination of different degrees of freshness is not simply based on the glossiness perception.

## Experiment 2

**Figure 4:**
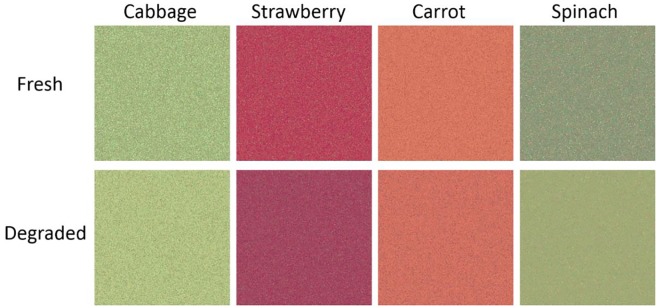
Stimuli used in Experiment 2.

Previous studies showed that infants appear to prefer stimulus images with high contrast (Slater, 1995), high saturation (Zemach et al., 2007), and particular contrast polarity (Dannemiller and Stephens, 2001). Therefore, it is possible that the infants may have preferred the images of fresh cabbage due to specific low-level image attributes, rather than visually apparent freshness. In Experiment 2, we tested if infants’ preference for fresh cabbage image stemmed from lower level image attributes (e.g., contrast, chromaticity). We performed experiments with infants aged 7–8 months using stimuli created by randomizing the pixels of the images used in Experiment 1 (Figure 4). These pixel-randomized images prevented infants from recognizing the object from the images but preserved the stimuli’s low-level image attributes. If low-level image attributes caused infants’ discrimination between fresh and degraded vegetables, the infants’ stimulus preferences would remain the same as in Experiment 1 after the stimuli’s pixels were randomized.

### Method

#### Participants

Participants were 17 infants aged 7–8 months (four males, 13 females; mean age = 224 days, range = 195–251 days). Three additional infants participated but were excluded from the analysis due to failure to complete the test (*n* = 2) or side bias of >90% (*n* = 1). Participants’ parents provided a written indication of informed consent.

#### Stimuli

We randomly redistributed the pixels in each of the original stimuli. This operation preserved the stimuli’s low-level image attributes and image statistics because only the pixels’ arrangement was changed. The pixel-randomized stimuli representing fresh and degraded foods were presented side by side on the monitor as in Experiment 1.

#### Apparatus and Procedure

The apparatus and procedure were the same as those used in Experiment 1.

### Results and Discussion

**Figure 5:**
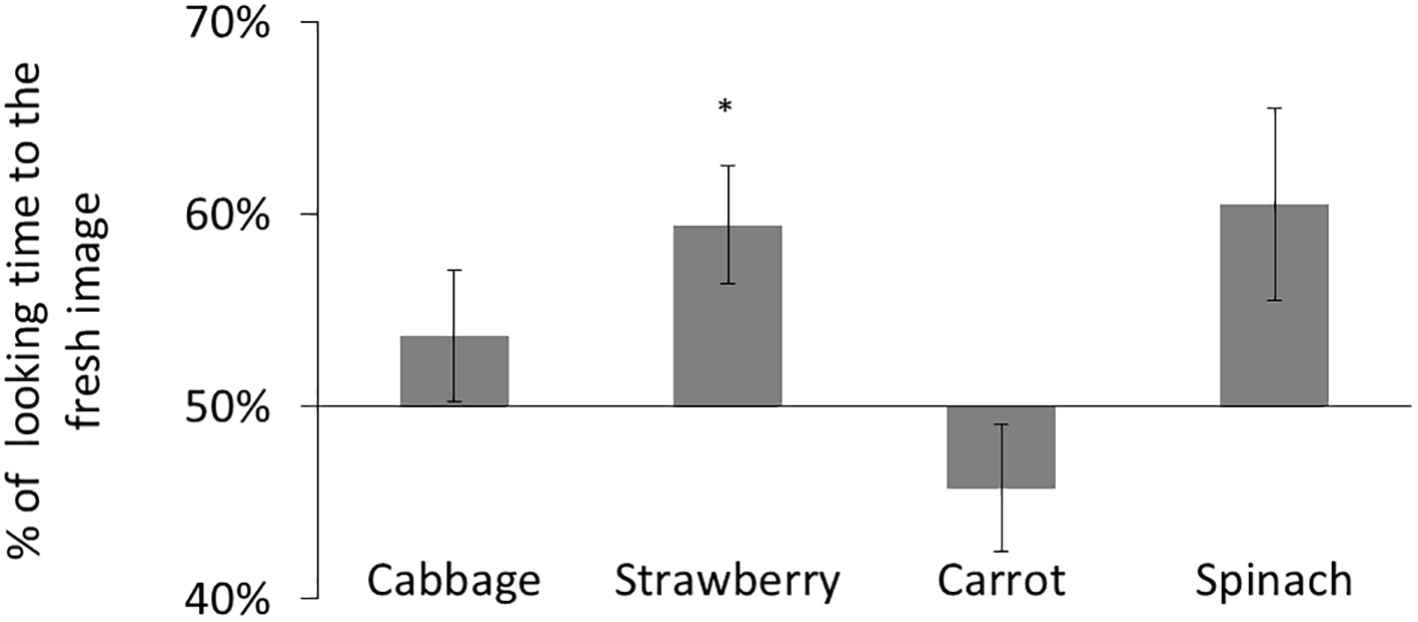
Result of Experiment 2. Mean percentage of looking time for fresh images. The error bars represent SE. Asterisks indicate the significance level of statistical differences: ^*^p < 0.05.

Participants’ mean total looking time across eight trials was 68.4 s (53.8% of the total trial duration). We calculated preference scores for each participant (i.e., the ratio of time looking at the fresh image versus total looking time; Figure 5). Two-tailed *t* tests comparing with chance indicated that the preference for fresh cabbage observed in Experiment 1 was absent [*t*(16) = 1.57, ns]. This result indicated that low-level image attributes did not cause infants’ preference in Experiment 1.

Participants showed a significant preference for the fresh strawberry stimuli [*t*(16) = 3.14, *p* < 0.05, Bonferroni-corrected, *d* = 0.75] but not for the carrot or spinach stimuli [*t*(16) = 0.09, ns; *t*(16) = 2.72, ns, respectively]. The participants may have preferred the fresh strawberry stimulus because infants tend to prefer high chromatic saturations [Zemach et al., 2007; differences in chromatic saturation (Table 1; Δ*C* in CIE L*u*v* color space) between fresh and degraded images: Δ*C* = 1.15, cabbage; Δ*C* = 6.21, strawberry; Δ*C* = 1.62, carrot; Δ*C* = −3.53, spinach]. Zemach et al. (2007) tested the infants’ chromatic saturation preference by using uniform color patches. In the present study, as long as the pixels of the original images were shuffled, these images looked more like uniform color patches without any texture, especially considering the poor spatial resolution of the infants. Therefore, the preferences were dominated by the chromatic saturation, and the infants showed the preference for the fresh strawberry stimuli. However, in Experiment 1, the high level information abstracted from the texture and the 3D structure could be more attractive in the degraded strawberry. Therefore, cancellation of the reverse preferences did not significantly affect the preference for strawberries.

## Experiment 3

Adults’ studies, which investigated the relationship between luminance distribution and freshness perception, indicated that the visual freshness perception is partly determined by skewness of images’ luminance distribution (Wada et al., 2010; Arce-Lopera et al., 2012, 2013, 2015). In adult observers, evaluations of freshness appear based on the information contained in the luminance rather than in the chromaticity. If infants use the same optical cue to discriminate the fresh and degraded cabbage image, they would show similar preference for the achromatic image that had the same skewness of images’ luminance distribution. In Experiment 3, we used achromatic version of stimuli used in Experiment 1 to test if luminance cues are sufficient for freshness perception in infants. Furthermore, if this extra experiment replicates the result in Experiment 1, it could confirm that the result in Experiment 1 was not simply a Type I error.

### Method

#### Participants

Participants were 17 infants aged 7–8 months (six males, 11 females; mean age = 229 days, range = 205–249 days). Six additional infants participated; however, they were excluded from the analysis due to fussiness (*n* = 4) or side bias of >90% (*n* = 2). Participants’ parents provided a written indication of informed consent.

#### Stimuli

The stimulus images from Experiment 1 were converted to grayscale using a photo editing grogram.

#### Apparatus and Procedure

The apparatus and procedure were the same as in Experiment 1.

### Results and Discussion

**Figure 6:**
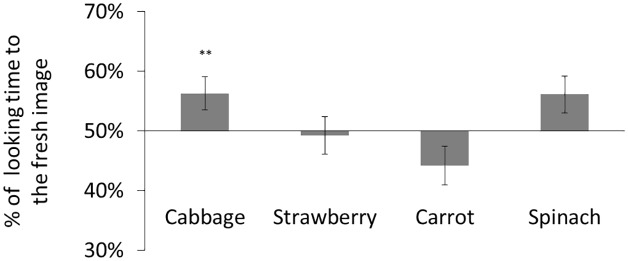
Result of Experiment 3. Mean percentage of looking time for fresh images. The error bars represent SE. Asterisks indicate the significance level of statistical differences: ^**^p < 0.01.

Participants’ mean total looking time across eight trials was 68.4 s (58.5% of total trial duration). We calculated a preference score for each participant (Figure 6). Two-tailed *t* tests comparing with chance indicated that participants significantly preferred the fresh cabbage stimulus [*t*(16) = 3.11, *p* < 0.01, Bonferroni-corrected, *d* = 0.75]. No other significant preferences were identified [strawberry: *t*(16) = 0.25, ns; carrot *t*(16) = 1.57, ns; spinach *t*(16) = 1.93, ns]. This result indicated that, like adults, luminance is sufficient for infants’ perception of freshness. More importantly, this experiment replicated the finding in Experiment 1, suggesting the fresh cabbage preference shown in Experiment 1 was not simply a Type I error.

## General Discussion

This study examined perceptions of freshness among infants aged 5–8 months. We found that infants aged 7–8 months, but not aged 5–6 months, discriminated between images of fresh and degraded cabbage (Experiment 1). This discrimination was not based on a spontaneous preference for low-level image attributes (Experiment 2), and achromatic information appeared sufficient for infants to perceive freshness (Experiment 3). These findings indicated a possibility that infants aged 7–8 months who have only little experience in tasting may have an ability to discriminate the different degrees of freshness.

Recent results implied that human and non-human primates evaluate freshness using luminance distributions (Wada et al., 2010; Arce-Lopera et al., 2012, 2013, 2015; Imura et al., 2016). We speculated that infants aged 7–8 months use this same cue to discriminate between different degrees of freshness. First, this finding was in line with a recent infant study in which 9-month olds showed sensitivities to natural image statistics (Balas and Woods, 2014). Second, even when achromatic images were presented in Experiment 3, infants aged 7–8 months tended to look longer at the fresh cabbage image. This result indicated that infants use only luminance cue to discriminate between different degrees of freshness. As mentioned in section “Introduction,” this study aimed to investigate the ability to discriminate between different degrees of freshness in infants, rather than to test whether infants have a grasp of the concept of “freshness.” Our results demonstrated that 7–8-month olds already have this visual ability. In line with the present study, previous studies reported that food-related perception develops early in life. Infants aged 6–8 months appear to prefer correctly, rather than incorrectly, colored images of fruits (Kimura et al., 2010), and begin to recognize olfactory-visual congruency around the same age (Wada et al., 2012). These findings collectively indicated that infants can integrate multiple cues to recognize natural foods; they have a rudimentary perceptual foundation to develop the concept of “freshness” and knowledge about food, which may shape learned food preferences (Birch, 1999), even though they have little chance to taste any food before weaning.

The infants only succeeded in cabbage but failed in other foods, and this indicated that infants’ ability to judge freshness appears rudimentary and to depend on strong cues in the stimuli. A possible explanation is that the familiarity with the foods would affect the visual preference showed by infants. To investigate the familiarity with the foods, we assumed that the familiarities are correlated with the shipments in the Japanese market. In Japan, more than 808,700 tons of cabbages were distributed for wholesale trade in 2015, nearly twice amount of carrots (428,300 tons), six times amount of strawberries (145,200 tons), and 15 times amount of spinaches (54,700 tons; Ministry of Agriculture, Forestry and Fisheries, 2018). These data indicated that it was higher frequency of participant exposure to cabbages during the daily life, and this higher familiarity with cabbages should be a factor enhancing the freshness preference in the infants aged 7–8 months.

The present results indicate that infants aged 7–8 months can discriminate between images of fresh and degraded cabbage, and this discrimination probably stems from the perceived freshness. However, it also remains possible that infants use visual attributes other than luminance distribution (e.g., highlight patterns) to evaluate freshness. Accordingly, the present results represent initial findings regarding freshness perception in infants, rather than conclusive evidence. Future research should conduct further direct examination of the cues infants use to assess freshness and test whether infants can categorize the concept of freshness from different kinds of food.

## Ethics Statement

All experiments in the present study were approved by the Ethics Committee of Chuo University and were conducted in accordance with the Declaration of Helsinki. Participants’ parents provided a written indication of informed consent.

## Author Contributions

JY performed testing and data collection. JY performed the data analysis and interpretation under the supervision of KO, SK, and MY. JY drafted the manuscript. KO, SK, and MY provided critical revisions. All authors contributed to the study design and approved the final version of the manuscript for submission.

### Conflict of Interest Statement

The authors declare that the research was conducted in the absence of any commercial or financial relationships that could be construed as a potential conflict of interest.
